# The Effects of Feed Particle Size and Floor Type on the Growth Performance, GIT Development, and Pododermatitis in Broiler Chickens

**DOI:** 10.3390/ani10081256

**Published:** 2020-07-24

**Authors:** Amr Abd El-Wahab, Jan-Philip Kriewitz, Julia Hankel, Bussarakam Chuppava, Christine Ratert, Venja Taube, Christian Visscher, Josef Kamphues

**Affiliations:** 1 Department of Nutrition and Nutritional Deficiency Diseases, Faculty of Veterinary Medicine, Mansoura University, Mansoura 35516, Egypt; amrwahab5@mans.edu.eg; 2 Institute for Animal Nutrition, University of Veterinary Medicine Hannover, Foundation, Bischofsholer Damm 15, D-30173 Hanover, Germany; jan-philip.kriewitz@tiho-hannover.de (J.-P.K.); julia.hankel@tiho-hannover.de (J.H.); Bussarakam.Chuppava@tiho-hannover.de (B.C.); christine.ratert@tiho-hannover.de (C.R.); josef.kamphues@tiho-hannover.de (J.K.); 3 BEST 3 Geflügelernährung GmbH, D-27239 Twistringen, Germany; V.Taube@best-3.de

**Keywords:** broiler, floor design, diet, performance, pododermatitis

## Abstract

**Simple Summary:**

The use of dietary structural components like whole grains together with using different floor designs is of great interest in terms of alterations of the digestive tract, level of feed intake, growth performance and foot pad health in broilers. The current study tested the impact of feed particle size and various floor systems (litter only; litter with floor heating; 50% or 100% slatted floors) on the abovementioned parameters in broiler chickens. A finely ground diet reduced gizzard and pancreas weights compared to a coarsely ground diet. A coarse diet led to a marked higher body weight. No effect of feed particle size on foot pad health was observed. Housing broilers on different flooring designs showed no effect on gizzard and pancreas weights. Using a fully slatted floor led to a higher body weight while having no effect on reducing the incidence of foot pad dermatitis.

**Abstract:**

The aim of the present study was to evaluate the effects of feed particle size and flooring designs on organ traits, performance and pododermatitis in broilers. A total of 480 broilers (Ross 308) of both sexes were randomly assigned to two feeding groups (finely or coarsely ground pelleted diets; with addition of 5% to 10% intact wheat in coarsely diets) and four different housing systems (litter; litter with floor heating; partially or fully slatted floor) with three subgroups each. A coarse diet increased the final gizzard and pancreas weights (*p* < 0.001) while decreasing the risk of *Isthmus gastrici* dilatation compared to a fine diet (*p* < 0.001). Broilers fed a coarse diet displayed an increased final body weight (*p* = 0.023) and led to a favourable feed conversion ratio. Final body weight was the highest (*p* < 0.001) for birds housed on partially or fully slatted floor. Housing birds on litter with floor heating showed the lowest pododermatitis scoring (*p* < 0.001). It seems to be favourable to use coarse diets for organ development, whereas slatted floors seem to foster enlargement of the *Isthmus gastrici*. Increasing growth performance was possible both when using coarse diets or slatted floors.

## 1. Introduction

In recent years, the interest in feed particle size has increased and the feed industry continues to search for ways to optimise and improve feed utilisation, animal health including that of foot pads and growth performance [1]. Various technologies for treating of feed ingredients and compound feeds are available for the feed industry. Reducing grain particle size is the classical approach to follow for improving pellet quality as well as allowing a greater interaction with digestive enzymes because of an increased grain surface area [2] which consequently improves digestion. A standard morphology of the proventriculus, working normally with the gizzard, may cause digesta to remain for a longer period in the upper tract and then to move through the intestinal tract more rapidly. This process may allow enzymes to hasten the digestion [3]. However, the normal digestive processes may be disrupted by dilatation of the proventriculus. It is thought that a large particle size aided by some structural components is beneficial to gizzard functions and gut development. Nevertheless, reduced grain particle size can lead to poor gizzard development and, hence, decrease feed utilisation [4]. The gizzard plays a dynamic role in the digestive system of poultry, responding rapidly to any changes in the diet [5]. A large and well-developed gizzard is able to grind feed particles more thoroughly [6], to elevate pancreatic enzyme secretion [4], to increase proteolysis by pepsin and trypsin, to improve gastrointestinal tract motility and to improve nutrient digestibility [7]. Furthermore, Witte [8] stated that feeding broilers on coarser particles is associated with higher gizzard weights due to the enlargement of the whole organ rather than an increased tunica muscularis layer thickness. However, to date, there is a controversial debate regarding the optimum grain particle size in poultry.

It is necessary to optimise the health of poultry by focusing on dietary strategies as well as management conditions. The influence of housing and management regarding animal health and welfare in poultry is well documented [9]. Litter quality has attracted considerable attention, on the one hand, due to the fact of its use as a bedding material for most commercial poultry production [10] and, on the other hand, as it provides an aspect of animal welfare like pecking and scratching [11]. It is well known that litter is used to decrease birds’ contact with excreta and that it helps to absorb surplus moisture [12]. Moreover, with bad litter quality, the birds develop pododermatitis which causes pain, an issue of animal welfare [13]. Variable factors could affect litter quality markedly which are either linked to diets or to management and housing [14,15]. Different studies have been conducted to improve litter quality and, hence, reduce the severity of pododermatitis such as using different litter materials or using floor heating [16]. Poultry farming with slatted flooring has recently been used in broilers [17,18]. A plastic slatted floor is very durable and cost-effective, does not deteriorate or require replacement, is easy to install and clean and may decrease incidents of foot pad injuries [12]. This trend has been developed to reduce the contact between birds’ feet and their excreta in some parts of the world. However, it cannot be neglected that the floor designs per se could have a negative effect on animal welfare. Necrotic and ulcerative lesions of the foot pads can be very painful and cause stress [19] and could be a gateway for pathogenic organisms causing a decline in the animals’ general health condition. Reduced performance, including unfavourable feed conversion ratio (FCR) and lower final body weight, is the expected outcome [20].

The objective of this study was firstly to investigate the effects of feed particle size (finely and coarsely ground diets) on the development of the digestive tract, growth performance and pododermatitis in broiler chickens. Secondly, to test whether perforation has also an effect on these parameters to evaluate if perforation is highly frequented and whether stressed areas with commonly bad litter quality in practice could be a solution for the future. Finally, the diet factor is more relevant than housing on the gastrointestinal tract (GIT) development; however, it could be interesting to investigate an interaction between feed and floor type, especially when missing the required/sufficient dietary physical size, on the feed intake and GIT development as the litter material might be act as a substituting factor.

## 2. Materials and Methods

The experiments were performed in accordance with German regulations and approved by the Ethics Committee of Lower Saxony for Care and Use of Laboratory Animals (LAVES, Niedersächsisches Landesamt für Verbraucherschutz und Lebensmittelsicherheit; reference: 33.12-42502-04-16/2085).

### 2.1. Experimental Design, Animals and Housing

The experimental study was conducted with 480 birds of one-day-old broiler chickens (Ross 308) of both sexes. The animals were obtained from a commercial hatchery. The broiler chickens were housed in floor pens littered with wood shavings (GOLDSPAN^®^, Goldspan GmbH and Co. KG, Goldenstedt, Germany) in groups of approximately 60 birds for the first week of life. Litter material was kept dry before the experiment was started by replacing the upper layers of dirty litter with fresh litter. At day four of life, the birds were vaccinated against Newcastle disease using Nobilis ND Clone 30^®^ (MSD Animal Health Innovation GmbH, Unterschleissheim, Germany). The environmental temperature was gradually reduced from about 35 °C for the one-day-old birds to about 20 °C by day 36/37 according to the rearing conditions of Aviagen [21]. The relative humidity was about 72.3% ± 13.2 throughout the trial. Lights were continuously on for the first three days of life and the photoperiod from day four days onwards was 16 h of light and 8 h of darkness.

At the beginning of the second week, birds were allocated randomly to groups depending on flooring (four variants) and feed (two variants). In total, eight groups were formed, with three subgroups of 20 birds each in experimental pens (1.20 × 0.80 m). The birds had ad libitum access to fresh, clean water via nipple drinkers (Big Dutchman International GmbH, Vechta, Germany). It has to be mentioned that at days 10–14 of life, the birds were treated with antibiotic Enrofloxacin (Baytril^®^ 10%, Bayer Vital GmbH, Leverkusen, Germany) in the drinking water (10 mg Enrofloxacin/kg BW/day) due to the resistance against antibacterial agents in commensal *Escherichia coli* depending on different flooring [22,23]. At day 23 in each subgroup, eight birds were selected randomly and dissected (*n* = 24). Thereafter, the remaining 12 birds were dissected in each subgroup at day 36/37. The stunning method was performed in accordance with Annex I of Council Regulation (EC) No. 1099/2009, ‘Chapter I, Methods, Table 1-Mechanical Methods, No 6’; a percussive blow to the head was made, followed by bleeding the animals. Thereafter, the gastrointestinal tract and the internal organs were removed, whereas the feathers were not.

**Figure 1:**
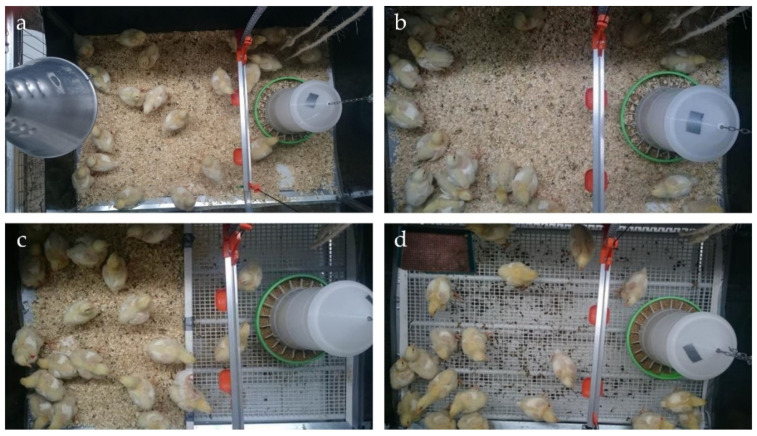
Characteristics of flooring designs: (**a**) entire floor pen covered with litter; (**b**) floor pen covered with litter and floor heating; (**c**) partially slatted flooring; (**d**) fully slatted flooring with sand bath (photo: ©Kriewitz/TiHo).

Different flooring systems were established to obtain different contact levels of birds’ foot pads to the excreta (Figure 1). The first group (L^+^H^−^) was kept on wood shavings (whole floor pen covered with litter); the second group (L^+^H^+^) was kept on wood shavings but also with floor heating (litter surface temperature 30 °C). An electric floor heating system supplied with an adjuster to control the temperature was used (Sauerland GmbH, Paderborn, Germany). The birds in the aforementioned two groups had full contact with the mix of wood shavings and excreta during the entire study period. The third group (L^+/−^H^−^) was housed in a floor pen that was divided into two equal parts consisting of 50% wood shavings on the scratching area and 50% plastic slatted floor on the feeding area. The slatted floor consisted of holes (15 × 10 mm) and bridges (plastic covered steel; width 3.5 mm; Big Dutchman International GmbH, Vechta, Germany). The fourth group (L^−^H^−^) was housed completely on plastic slatted floor with a sand bath (900 cm^2^). The sand bath contained about 300 g of sand at the beginning of the experiment at day eight, approximately 100 g of sand being added every two days thereafter throughout the experiment. Broilers housed on fully slatted flooring had the lowest contact intensity with litter except possibly inside the sand bath.

### 2.2. Diets

All birds were fed ad libitum with commercially produced pelleted diets from a local feed manufactory via one hanging-type feeder (Crown Chicken Ltd., Norwich, UK). All diets were based on wheat, soybean meal and yellow corn in addition to rapeseed meal during the grower phase; rapeseed meal and triticale were included during the finisher phase. Feeds were milled completely by hammer mill (finely ground diet = F; coarsely ground diet = C). In group C, the wheat was added in parts intact to the feed at the time of loading into the truck at the feed company. Therefore, in the experimental group, the starter diet contained 3% (first week), the grower diet 5% (second week) and the finisher diet 10% intact wheat up to the end of trial (d 15-d 36/37; Table 1).

#### 2.2.1. Wet Sieve Analysis

For determining particle size distribution within the six different diets, a wet sieve analysis was performed according to Wolf et al. [24]. The sieves (sieve tower consisting of eight screen layers with the following mesh sizes in mm: 3.15; 2.0; 1.4; 1.0; 0.8; 0.56; 0.4; 0.2; Retsch GmbH, Haan, Germany) were initially placed in hot air at 103 °C, and after cooling in a desiccator, weighed separately. A feed sample of approximately 30–50 g was soaked for one h in one L of water to ensure adequate hydration. Then the suspension was rinsed with water (10 L) at a standardised pressure through the sieve tower. The wet sieves were dried overnight at 103 °C and weighed after cooling in a desiccator. The weight of particles retained on each sieve was then expressed as a percentage of the total dry matter (DM). The parts of the sample which had been washed out through the smallest screen layer (finest and dissolved constituents such as sugars and electrolytes) were calculated by subtraction.

#### 2.2.2. Particle Size

The particle size distribution of the diets is summarised in Table 2. The percentage of particles greater than 2 mm differed markedly between finely and coarsely ground diets in each dietary phase. About 17.9%, 11.6% and 9.66% of particles were >2 mm in finely ground diets for starter, grower and finisher diets, respectively. In contrast, in coarsely ground diets the percentage of particle distribution >2 mm was 23.3%, 27.0% and 29.5% for starter, grower and finisher diets, respectively. Moreover, the percentage of particle size <0.2 mm was 25.6% in finely ground diet (starter phase) versus 25.0% in coarsely ground diet (starter phase).

### 2.3. Chemical Composition

The composition of the diet was formulated according to the requirements of each fattening phase of broiler chickens (Table 3). The various diets were analysed in accordance with the official methods of the Association of German Agricultural Analytic and Research Institutes [24]. The DM content was determined mathematically by weighing before and after drying the samples at 103 °C. The crude ash content was detected by weighing the samples before and after combustion in the muffle furnace at 600 °C. The Soxhlet apparatus was used to measure the crude fat content using a standard protocol and the crude fibre content was determined through washing the samples in dilute acids and alkalis. Moreover, the Dumas incineration method (Vario Max, Elementar, Langenselbold, Germany) was applied to get the total *N* content. Sugar in the samples was analysed by using the Luff–Schoorl method, while the atomic absorption spectrometry was used to analyse some minerals (Unicam Solaar 116, Thermo, Dreieich, Germany). Ion-exchange chromatography (AA analyzer LC 3000, Biotronic, Maintal, Germany) was used to analyse amino acids. Finally, determining the starch content of the diets was accomplished polarimetrically (Schmidt und Haensch GmbH & Co., Berlin, Germany).

### 2.4. Growth Performance and Isthmus Gastrici Scoring

The individual body weight (BW) was measured at days 8, 23 and 36/37. It has to be mentioned that the rearing phase began with the delivery at 1st d of life and ended with the transfer to the experimental stable (d 8), i.e., the BW measured at d 8 was after the completion of a seven-day rearing period. At dissection (day 36/37) carcass weights (without head and feet) were taken, too. Feed and water intakes were determined at the subgroup level. The feed conversion ratio was estimated on the basis of feed consumed throughout the experimental period as well as body weight gain of the birds. The water-to-feed-intake ratio was calculated by divided the total water intake to the total feed consumed throughout the experimental period at each subgroup level.

**Figure 2:**
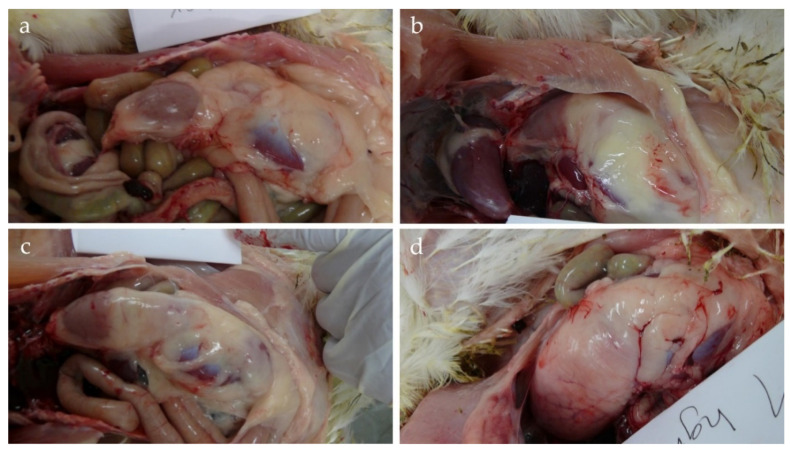
Scoring of *Isthmus gastrici*: (**a**) Score 0 = anatomically correct; (**b**) 1 = slight dilatation; (**c**) Score 2 = moderate dilatation; (**d**) Score 3 = high dilatation (photo: ©Kriewitz/TiHo).

The abdominal cavity was opened carefully. Thereafter, the *Isthmus gastrici* was assessed macroscopically in accordance with Witte [9] as follows: Score 0 = normal; Score 1 = slight dilatation of the gastric, glandular, and *Isthmus gastrici* are clearly differentiable; Score 2 = distinct dilatation of the *Isthmus gastrici*, constriction however still recognisable; Score 3 = *Isthmus gastrici* is completely absent (Figure 2). Gizzard and pancreas were detached and weighed individually as a relative to the BW.

### 2.5. Litter DM and Pododermatitis Scoring

Litter samples for measuring the DM content were collected at days 9, 14, 21, 28, 35 and 37 from the right and left areas of the pen. At each area, a sample (50 g) from three sites (two peripheral samples and one central one) over the entire bedding height was punched out from the full depth of the litter using a cup with a diameter of 5 cm. Samples were dried at 103 °C for the time needed to reach a constant weight [25]. The external examination of foot pads was performed for all birds at the beginning of the trials at day 8, then at days 23 and 36/37. The signs of foot pad dermatitis were recorded only at the central plantar area on a seven point scale (0 = normal skin; 3 = small black necrotic areas; 7 = over half of the foot pad is covered with necrotic scales) in accordance with Weiss [26], modified from Mayne et al. [14].

### 2.6. Statistical Analyses

The statistical analysis was performed using the Statistical Analysis System for Windows, the SAS^®^ Enterprise Guide^®^, version 9.3 (SAS Institute Inc., Cary, NC, USA). Organ traits, BW, and pododermatitis scores were analysed at the level of the individual animal, further values, i.e., FI, FCR and W/F at box level. For most parameters, mean values as well as the standard error of the mean (SEM) were calculated. Pododermatitis scores were determined by forming the mean of the scores of both feet of each animal. A test for normal distribution was performed using the Shapiro–Wilk test. Therefore, data of organ traits, DM content of litter, and pododermatitis scores were analysed by the Kruskal–Wallis test, followed by a post-hoc Dunn’s test for multiple pairwise comparisons. Normally distributed data (performance) were analysed by two-way analysis of variance (ANOVA) with flooring and feed as independent factors. Differences regarding the scoring of *Isthmus gastrici* depending on flooring and feed were analysed using the chi-square homogeneity test. Differences with a significant level of *p* < 0.05 were considered significant.

## 3. Results

### 3.1. Organ Traits

Relative weights of the gizzard and pancreas in broilers are reported in Table 4 and Table 5. Significant differences were found in gizzard and pancreas weights at days 23 and 36/37 at group level with combined factors (Table 4). At group level, all broilers fed coarsely ground diets displayed significant differences in relative gizzard weight at both dissection days (*p* < 0.001; Table 4). There was no significant effect in the relative weights of gizzard and pancreas in the birds depending on flooring designs either at d 23 or d 36/37 (Table 5). The weight of the gizzard of broilers at d 23 or d 36/37 fed coarsely ground diet were significantly (*p* < 0.001) higher than those fed a finely ground diet (Table 5). Also, the broilers fed a coarsely ground diet had a significantly (*p* < 0.001) higher pancreas weight than those fed a finely ground diet at both dissection days.

The results of *Isthmus gastrici* scoring in broilers are shown in Table 6. There was no significant difference in *Isthmus gastrici* scoring at d 23 of life between broilers fed finely or coarsely ground feeds (*p* = 0.055). Nevertheless, at d 36/37, the scoring of *Isthmus gastrici* was affected significantly by feeding finely or coarsely ground feeds. By rearing birds on different flooring designs, non-significant effects were observed in *Isthmus gastrici* scoring at d 23 of life (*p* = 0.339). However, the *Isthmus gastrici* scoring was significantly affected by housing birds on different flooring designs at d 36/37 (*p* = 0.019).

### 3.2. Growth Performance

The results of growth performance in broilers are shown in Table 7 and Table 8. A marked interaction of flooring and feed was observed on FI (*p* = 0.042; Table 7). The highest FI was noted for broilers housed on fully slatted floor and fed a coarsely ground feed, while those reared on litter with floor heating and fed a finely ground diet had the lowest FI (115 versus 101 g; Table 7). Daily feed intake (FI) was significantly affected by housing broilers on different flooring designs (*p* < 0.001). Also, during the fattening experiment the highest significant (*p* < 0.001) FI was observed after feeding coarsely ground diet (Table 8). There was not a significant flooring by feed interaction in the water to feed intake ratio during the entire rearing period. In general, broilers kept on litter with floor heating were characterized by a significantly higher water to feed ratio compared to other flooring designs (Table 7). A significant difference was observed in the water to feed intake ratio for broilers housed on different flooring designs (*p* < 0.001; Table 8). However, it was noted that the type of diet fed to broilers had no significant effect on the water to feed intake ratio (*p* = 0.435; Table 8).

There was no significant flooring by feed interaction in the case of BW recorded throughout the experimental period. Interestingly, at the group level (Table 7), birds housed on partially slatted floor and fed coarsely ground diet had the highest final BW (2514 g), whereas those housed on litter with floor heating and fed a coarsely ground diet had the lowest BW (2191 g). There was also no significant difference in BW either at d 8 or d 23 of life between broilers housed in different flooring designs (*p* = 0.295; *p* = 0.299, respectively). Nevertheless, at d 36/37, broilers kept on fully slatted flooring had a significantly (*p* < 0.001) higher BW (Table 8). Broilers fed a coarsely ground diet had a significantly (*p* < 0.001) increased BW compared to those animals fed finely ground diet at d 23. Similarly, at d 36/37, the BW of broilers fed a coarsely ground diet was higher compared to those fed a finely ground diet (*p* = 0.023; Table 8). There was not a significant (*p* = 0.214) flooring by feed interaction in FCR. Broilers fed coarsely ground diet and housed on only litter or with floor heating or fully slatted floor had a worsened FCR (1.49; Table 7). A significant difference (*p* = 0.155) between groups kept on different flooring designs related to FCR was not noted (Table 8). The FCR was more favourable for birds fed finely ground diet than those fed coarsely ground diet (*p* < 0.001; Table 8).

### 3.3. Litter Quality and Pododermatitis

The data on DM content of litter and pododermatitis scoring are presented in Table 9. A significant difference in DM content of litter among different flooring designs was observed (*p* < 0.001). No significant differences were observed concerning DM content in broilers fed different feeding particle sizes of diets (*p* = 0.461). Interestingly, there was significant flooring by feed interaction for DM content in litter (*p* < 0.001). The highest value of litter DM content was found in groups housed on litter with floor heating regardless of the type of feed.

At the beginning of the trial, all foot pads of the birds were healthy. However, at the end of the trial (d 36/37) there were significant differences (*p* < 0.001) in foot pad lesions for those broilers kept on different flooring designs (Table 9). No significant differences were observed in pododermatitis scores for broilers fed different feeding forms (*p* = 0.391). There was a significant flooring by feeding interaction in pododermatitis scores (*p* < 0.001). Broilers housed only on wood shavings and fed a finely ground diet had significantly higher FPD score than those fed coarsely ground diet (0.88 vs. 0.63). However, broilers kept on wood shavings with floor heating and fed a finely ground diet had the lowest pododermatitis score, while those birds housed on partially slatted floor had the highest pododermatitis score (0.60 vs. 1.08).

## 4. Discussion

The present study aimed to evaluate the benefits and drawbacks of different flooring designs and feed particle sizes on digestive tract development, performance and foot pad health in broilers.

### 4.1. Impact of Flooring and Feed on Organ Traits

The outcome of the current study showed no significant effects of flooring designs on gizzard and pancreas weights. These results are in agreement with findings of Toghyani et al. [27] who reported that the gizzard weight was not significantly affected by reared broiler chickens for 42 d with or without litter but tended to decrease in broilers reared on no litter and increase in wood shaving. However, several reports have indicated that dietary insoluble fibre stimulated gizzard function [28]. There is clear evidence that the particle size and the physical form of the diet have an effect on the development of the birds’ digestive system, which is also accompanied by changes in physiological functions [29]. Moreover, several studies have shown some variations in results, mainly due to the different experimental conditions. It is well defined that the gizzard is a dynamic organ which consequently responds rapidly to dietary changes [5]. However, the gizzard size may increase to over 100% of its original size when structural components are added to the diet [4]. In the present study, a coarsely ground diet significantly increased the gizzard weight of broilers compared to a finely ground diet. The average relative weight of gizzard at d 23 for broilers fed coarse ground diets was approximately 22.65 and 11.50 (g/kg BW) versuus approximately 14.48 and 8.73 (g/kg BW) for those fed finely ground diet for day 23 and 36/37, respectively. Similar results were also observed by Svihus et al. [30] who found that feeding coarsely wheat led to an increased gizzard weight compared to finely wheat (17.5 vs. 14.9 g/kg BW). Additionally, Péron et al. [31] observed that gizzard weight was higher for birds fed coarsely wheat diets than those fed finely wheat diets (15.0 vs. 13.0 g/kg BW). Singh et al. [32] reviewed ten studies involving 18 paired sets of observations in which the relative gizzard weight was increased by an average of 44.0% (range from 18.2 to 100%). Truong et al. [33] reported that the 18.0% pre-pellet inclusion of whole grain increased relative gizzard weight by 13.0% (16.44 vs. 14.55 g/kg; *p* < 0.005). It could be that feeding bird whole grain led to an increased frequency of gizzard contraction to grind whole grain to fine parts and, hence, an increased gizzard weight. Nir et al. [34] suggested that coarser particles are better suited to poultry because of their stimulating effect on the gizzard size and gut motility. The stimulation of gut motility is an important effect of coarse particles and has been hypothesised to improve intestinal strength due to the greater muscular activity related to reverse peristalsis [35]. Moreover, a well-developed gizzard increases proteolysis by pepsin, trypsin and other endogenous proteases in the small intestine in addition to stronger reverse peristalsis contractions [36]. However, the results of studies related to the effects of particle size on gizzard development have been unsatisfactory. Preston et al. [2] found that a smaller particle size resulted in higher digestibility in poultry due to the greater interaction with digestive enzymes. Indeed, and according to Liermann et al. [37], the weight of the gizzard was negatively correlated to the feed intake (*r* = −0.678; *p < 0.*001) and to the BW gain (*r* = −0.575; *p* < 0.001). It was anticipated in previous studies that adding pre-pellet whole grain would not generate comparable increases in gizzard weight probably because steam-pelleting ‘crushes” whole wheat [38,39].

Specifically, in our study, coarsely ground diet significantly increased the weight of the pancreas compared to the finely ground diet (with an average of 1.57 versus 1.43 g/kg BW at d 36/37, respectively). Similar results were reported by Rougière et al. [40] who found that the weight of the pancreas of birds fed coarsely corn diet was about 2.37 g/kg BW compared to those fed finely corn diet (2.16 g/kg BW). They associated the higher organ weight with an increase in pancreatic secretion. As reported in study by Nir et al. [41], overfeeding resulted in a higher pancreas weight. This could be due to the increased secretory activity of the pancreas because of increased weight and/or activity of the gizzard. Nevertheless, Engberg et al. [42] observed that the pancreas weight for birds (d 42) fed either coarsely or finely wheat diets was identical (1.8 g/kg BW). The development of the gastrointestinal tract is a very complex procedure. The structure of a diet is a relevant factor even more than effect of litter material. Consequently, the marked effect of feed and floor interaction could be actually observed because of the dominant effect of feed structure on the development of gizzard and pancreas.

Especially in groups housed on fully slatted flooring the animals showed the highest prevalence of dilated changes in *Isthmus gastrici* at the end of the trial (scores 1 and 2; 30.0 and 21.4% respectively)*,* suggesting that the litter material had an obvious impact on the *Isthmus gastrici* dilatation. Riddell [43] observed that the young birds consumed some new litter material in addition to the feed in order to fulfil the needs for dietary structure or fibre. Interestingly, in the present study, broilers fed finely ground diet showed increases in *Isthmus gastrici* scores 1 and 2 (15.7 and 35.7%, respectively) compared to those fed coarsely ground diet. In agreement with our results, Taylor and Jones [3] found that the incidence of proventricular dilatation was reduced when whole grain was included into pelleted diets at 200 g/kg. It appears that in healthy birds whole grain inclusion in a ground compared to pelleted diet reduces proventricular dilatation and has positive effects on gut development and passage through the gastrointestinal tract [3,33]. Also, in the current study, no marked effect of flooring designs on the *Isthmus gastrici* was noted. It is suggested that massive expansion of the proventriculus (stretched rather than an actual increase in muscular tissue) may limit the available space for other internal organs [37]. The latter author indicated that the high feed intake of broilers fed a pelleted diet directly increases the risk of *Isthmus gastrici*. However, in the current study, feed intake is not the explanation for the degree of *Isthmus gastrici,* as broilers fed coarsely ground feed showed increases in feed intake but a low risk of *Isthmus gastrici*.

### 4.2. Impact of Flooring and Feed on Performance

Moreover, due to the increasing demand for processed poultry products there is a major concern to produce broilers with higher BW. To improve broiler performance, good management and optimum housing conditions along with perfect diet formulation should be practised. The performance data given by the breeding company on the genetics Ross 308 at d 36/37 (end of experiment) expect an average BW of 2192 and 2284 g according to Aviagen [21,44], respectively, while taking into account that d 0 in their data sheet is d 1. In the current study, the mean BW was 2376 g at d 36/37 and was therefore slightly above the expected level. The outcome of the current study showed that broilers in slatted flooring systems had a significantly higher final BW compared with those housed in litter systems. Specifically, the final BW was 76 g higher for broilers reared on fully slatted flooring than those reared on litter floor pens. Similar results were obtained from Almeida et al. [45] and Çavuşoğlu et al. [18] as broilers reared on plastic slatted flooring had relatively higher weight gains (+8.10% for the final BW) and a worse FCR than observed for chickens reared on only wood shavings. Similarly, Chuppava et al. [46] found that broilers fed identical diets and housed in a slatted flooring system had a significantly higher final BW compared with those provided with floor heating. The broilers housed on litter with floor heating showed the lowest feed intake, BW and the highest water/feed ratio. High ambient temperature during broiler fattening correlate negatively with feed consumption and positively with water intake, and they also have an unfavourable effect on FCR [47]. Thus, it was assumed that the described differences in the performance parameters were mainly due to the higher surface temperature. Littered flooring may offer potential probability for the broilers to peck the litter materials available on the ground resulting in lower feed intake which is not the case with slatted flooring [48]. Another possible explanation for increased feed intake and hence BW is that in slatted floor systems, the floor ventilation system improves air quality which led to reduced heat stress [49]. In contrast, using slatted floor did not affect BW in broilers compared to the littered floor [50].

In this study, the birds fed coarsely ground diet had higher BW with a difference of about 72 g than those fed finely ground diet. Moreover, the BW for birds fed coarsely ground diet (2412 g) was higher than for the data (2192 or 2284 g) given by Aviagen [21,44]. Decreased BW was clearly caused by the lower feed intake especially for broilers fed finely ground diet, which seemed to be mainly based on feed form a long with selective feed intake. Jones and Taylor [38] indicated that adding 200 g/kg of whole grain to the mix prior to pelleting resulted in consistent differences in gut morphology, which may contribute to an improvement in productive performance by way of health benefits. Moreover, the stimulation of gut motility an important effect of coarse particles, led to improved intestinal strength [35] with the overall result of improved nutrient utilisation. Possibly, increased BW of the broilers fed coarsely ground diet seems to be mainly based on feed intake. In contrast to studies by Svihus [4] who recommended including whole wheat in the diet to avoid over feed intake, this could not decrease the consumption when feeding a coarsely ground diet. After pelleting, if particle size differences persist in wheat-based diets, this results in improving feed efficiency of broilers [51]. On the other hand, no effect on performance was observed when pelleting did not show any differences in particle size distribution [30,42]. However, the effect on particle size distribution after pelleting regarding grain hardness needs further investigations.

### 4.3. Impact of Flooring and Feed on Litter and Pododermatitis

Litter quality is very important for broiler welfare since broilers spend all of their lives on litter material. Litter management is a crucial step for foot pad health. Moreover, the pododermatitis is considered as an indicator for housing conditions and the welfare of poultry [10]. It is well known that many different factors could affect pododermatitis. However, wet litter alone may be the cause [14]. Our study showed that broilers housed on floor heating had significantly lower pododermatitis scores compared to those reared without using floor heating. The temperature at the litter surface in the group with floor heating was about 30.4 °C with a difference of 2.70, 3.45, and 3.90 °C for the control, partially and fully slatted floor groups, respectively. Similar to our results, it was observed that young turkeys experimentally infected with coccidia housed on wet litter with floor heating had significantly lower pododermatitis scores compared to those reared without using floor heating, which could be attributed to the litter becoming much drier [52]. Contrary to the present results, Chuppava et al. [46] stated that birds housed in groups with floor heating (30.5 °C/16 h/d at litter surface) did not show significantly lower external pododermatitis scores compared to those housed in groups without floor heating. Another interesting point is the impact of using floor heating on the water-to-feed-intake ratio which was markedly higher in the present study. Furthermore, it was observed that the increases in water intake were additionally reflected to a progressive extent in litter moisture. In this study, despite enhanced water intake for birds housed in the group with floor heating, the litter moisture content was significantly lower than in other groups without floor heating. The outcome of this study shows no marked positive effects regarding slatted floorings on pododermatitis in broilers. Similarly, Almeida et al. [45] observed a slight tendency towards higher pododermatitis scores for birds housed on slatted flooring. However, Çavuşoğlu et al. [18] showed a lower frequency of pododermatitis in birds raised on slatted flooring.

The nutrition of birds is a major factor concerning the excreta moisture and litter quality [13] and consequently affects the incidence and intensity of pododermatitis. In the present study, there were no significant effects of the diet particle size on the DM content of litter and, hence, on the pododermatitis scores. To the best of our knowledge, no further studies have been performed regarding feeding broilers with different feed particle size combined with housing on dissimilar flooring designs on litter quality and health of foot pads. Nevertheless, it is assumed that the effects of different physical forms of diet could be mediated by changes in the water: feed intake ratio and its consequences on the DM content of the litter. Presumably, there are further effects due to the changes in the water release from the litter (drying of the litter upper layer); this property of the litter could also be influenced by the water binding capacity of undigested carbohydrates.

## 5. Conclusions

It seems that the most important factors affecting digestive tract, growth performance and foot pad health are feed (i.e., form, particle size). The data in this study revealed that at identical chemical composition, feeding diets to broilers with coarsely ground feed led to favoured gizzard development and higher body weight with no effect on foot pad health. Also, the results from this study suggest that floor designs had no effect on gizzard development, but when using fully slatted flooring, led to higher body weights. Unexpectedly, minimising contact between broilers’ feet and their excreta and/or litter quality by using fully slatted flooring resulted in unhealthier foot pads than the other flooring designs.

## Figures and Tables

**Table 1:** Composition of broiler commercial diets during three the growing phases (g/kg as fed).

Feedstuff	Dietary Phases (g/kg as fed)		
	Starter ^†^	Grower	Finisher
Wheat *	436	504	461
Soybean meal (42% crude protein)	322	268	251
Corn	145	114	90.0
Rapeseed meal (32% crude protein)	-	19.0	27.0
Triticale	-	-	90.0
Plant oil (soybean)	39.0	48.0	46.0
Animal fat (pig)	9.70	9.50	9.00
Calcium carbonate	17.0	15.0	10.0
Monocalcium phosphate	12.0	8.70	3.30
Sodium bicarbonate	1.80	1.90	1.90
Sodium chloride	1.80	1.40	1.40
Premix **	15.7	10.5	9.40

^†^ The starter diet was identical for all groups and contained 3% whole wheat. * Either coarse (5% or 10%) or fine ground for grower and finisher dietary phases. ** Composition (per kg) for starter diet: methionine hydroxy analogue 3.8 g; vitamin A 11640 IU, vitamin D3 4850 IU, vitamin E 58 mg, zinc 65 mg, iron 52.3 mg, manganese 106.6 mg, copper 14.5 mg, iodine 1.9 mg and selenium 0.29 mg; 6-phytase 485 FTU, 1,4-xylanase 1551 U, narasin 48.5 mg, nicarbacin 48.5 mg. ** Composition (per kg) for grower diet: methionine hydroxy analogue 3.3 g; vitamin A 9500 IU, vitamin D3 4750 IU, vitamin E 33 mg, zinc 47.5 mg, iron 19 mg, manganese 66.5 mg, copper 14.2 mg, iodine 1.9 mg and selenium 0.28 mg, 6-phytase 237 FTU, 1,4 -xylanase 1425 U, narasin 47.5 mg, nicarbacin 47.5 mg. ** Composition (per kg) for finisher diet: methionine hydroxy analogue 2.8 g; vitamin A 12000 IU, vitamin D3 5000 IU, vitamin E 35 mg, zinc 50 mg, iron 20 mg, manganese 70 mg, copper 15 mg, iodine 2 mg and selenium 0.28 mg, 6-phytase 225 FTU, 1,4-xylanase 1350 U, salinomycin-sodium 70 mg.

**Table 2:** Particle size distribution (% DM) in pelleted diets according to wet sieve analysis.

Wheat Grinding	Phase	Particle Size Distribution/mm (% DM)								
		>3.15	>2	>1.4	>1	>0.8	>0.56	>0.44	>0.2	<0.2
Fine	starter	1.74	17.9	20.2	12.3	5.62	7.21	4.40	5.03	25.6
	grower	0.18	11.6	19.5	14.7	5.00	6.32	3.00	3.40	36.3
	finisher	1.59	9.66	20.4	13.2	5.29	6.06	3.60	3.60	36.6
Coarse	starter	0.69	23.3	18.0	11.6	5.42	6.46	4.49	5.04	25.0
	grower	4.64	27.0	13.3	8.27	5.31	4.46	3.48	4.24	29.3
	finisher	3.53	29.5	13.0	15.6	4.35	5.53	3.26	2.93	22.3

**Table 3:** Chemical analyses of the experimental diets during the different fattening phases.

Parameter	Unit	Dietary Phases					
		Starter		Grower		Finisher	
		F	C	F	C	F	C
Dry matter	(g/kg as fed)	883	878	881	873	886	869
Crude protein	(g/kg DM)	266	247	228	232	229	235
Crude fat	(g/kg DM)	84.0	81.0	98.2	88.2	88.9	88.4
Crude fibre	(g/kg DM)	25.0	25.4	28.3	27.7	26.5	24.7
Crude ash	(g/kg DM)	66.7	61.7	52.6	55.6	49.4	48.5
Starch	(g/kg DM)	367	387	442	415	426	422
Sugar	(g/kg DM)	54.6	58.7	50.7	53.6	54.0	55.5
AMEn *	(MJ/kg DM)	13.8	13.8	15.0	14.3	14.4	14.5
Calcium	(g/kg DM)	11.1	10.3	8.72	8.71	7.09	7.00
Phosphorus	(g/kg DM)	7.72	6.69	5.32	6.45	5.62	5.48
Magnesium	(g/kg DM)	2.14	2.00	2.03	1.89	2.08	1.92
Sodium	(g/kg DM)	1.79	1.48	1.45	1.44	1.71	1.51
Potassium	(g/kg DM)	9.72	8.55	8.65	8.11	9.34	7.74
Chloride	(g/kg DM)	2.21	1.75	1.57	1.69	1.57	1.76
Sulphur	(g/kg DM)	3.85	3.83	3.17	4.07	3.06	3.49
Arginine	(g/kg DM)	17.8	16.9	15.2	15.7	15.2	15.2
Isoleucine	(g/kg DM)	10.4	9.91	8.54	9.32	8.58	9.45
Leucine	(g/kg DM)	19.6	17.0	16.1	16.2	16.5	11.5
Lysine	(g/kg DM)	17.3	15.7	13.7	14.1	13.7	12.6
Methionine **	(g/kg DM)	4.90	3.87	3.33	3.06	3.42	3.79
Threonine	(g/kg DM)	11.6	10.9	9.08	9.42	9.45	9.28
Valine	(g/kg DM)	11.8	11.2	10.1	10.5	9.66	10.8

F = Finely ground diet. C = Coarsely ground diet. * AME_n_ (per kg) = 0.1551 × % crude protein + 0.3431 × % ether extract + 0.1669 × % starch + 0.1301 × % sugar. ** Analysed as DL-methionine, whereas the methionine hydroxy analogue was added only in the premix.

**Table 4:** Relative mass of the gizzard and pancreas (g/kg BW ± SEM) of broilers at dissection days (flooring × feed).

Day	Organ (g/kg BW)	Flooring								*p*-Value
		L^+^H^−^		L^+^H^+^		L^+/−^H^−^		L^−^H^−^		
		Feed								Flooring × Feed
		F	C	F	C	F	C	F	C	
23	Gizzard	15.5 ^b^ ± 0.54	23.0 ^a^ ± 0.83	15.0 ^b^ ± 0.75	22.8 ^a^ ± 0.56	14.6 ^b^ ± 0.55	23.0 ^a^ ± 0.63	12.8 ^b^ ± 0.36	21.8 ^a^ ± 0.53	<0.001
	Pancreas	2.16 ^bc^ ± 0.08	2.74 ^a^ ± 0.07	2.13 ^c^ ± 0.08	2.45 ^ac^ ± 0.05	2.24 ^bcd^ ± 0.09	2.51 ^ab^ ± 0.07	2.06 ^d^ ± 0.08	2.45 ^ac^ ± 0.09	<0.001
36/37	Gizzard	9.08 ^b^ ± 0.30	11.9 ^a^ ± 0.49	9.04 ^b^ ± 0.35	11.4 ^a^ ± 0.44	8.91 ^b^ ± 0.30	11.6 ^a^ ± 0.53	7.92 ^b^ ± 0.20	11.1 ^a^ ± 0.31	<0.001
	Pancreas	1.44 ^ab^ ± 0.03	1.58 ^ab^ ± 0.06	1.37 ^b^ ± 0.05	1.53 ^ab^ ± 0.05	1.49 ^ab^ ± 0.05	1.61 ^a^ ± 0.06	1.41 ^ab^ ± 0.04	1.55 ^ab^ ± 0.06	0.009

^a,b,c,d^ Different subscripts within a row mark significant differences between the groups. F = Finely ground diet. C = Coarsely ground diet. L^+^H^−^ = Only litter. L^+^H^+^ = Litter and floor heating. L^+/−^H^−^ = Partially slatted floor. L^−^H^−^ = Fully slatted floor.

**Table 5:** Relative mass of the gizzard and pancreas (g/kg BW ± SEM) of broilers at dissection days (flooring, feed).

Day	Organ (g/kg BW)	Flooring					Feed		
		L^+^H^−^	L^+^H^+^	L^+/−^H^−^	L^−^H^−^	*p*-Value	F	C	*p*-Value
23	Gizzard	19.3 ± 0.84	18.9 ± 0.86	18.8 ± 0.89	17.3 ± 0.89	0.408	14.5 ^b^ ± 0.31	22.6 ^a^ ± 0.32	<0.001
	Pancreas	2.45 ± 0.08	2.29 ± 0.06	2.37 ± 0.06	2.26 ± 0.07	0.169	2.15 ^b^ ± 0.04	2.54 ^a^ ± 0.04	<0.001
36/37	Gizzard	10.4 ± 0.35	10.2 ± 0.33	10.2 ± 0.36	9.30 ± 0.29	0.154	8.74 ^b^ ± 0.15	11.5 ^a^ ± 0.23	<0.001
	Pancreas	1.51 ± 0.03	1.45 ± 0.04	1.55 ± 0.04	1.48 ± 0.04	0.252	1.43 ^b^ ± 0.02	1.57 ^a^ ± 0.03	<0.001

^a,b^ Different subscripts within row mark significant differences between the groups. F = Finely ground diet. C = Coarsely ground diet. L^+^H^−^ = Only litter. L^+^H^+^ = Litter and floor heating. L^+/−^H^−^ = Partially slatted floor. L^−^H^−^ = Fully slatted floor.

**Table 6:** Scoring of *Isthmus gastrici* (%) in broilers at dissection days.

Life Day	Score (%)	Flooring				Feed	
		L^+^H^−^	L^+^H^+^	L^+/−^H^−^	L^−^H^−^	F	C
23	0	67.4	83.7	81.3	72.9	70.2	82.5
	1	15.2	2.00	10.4	14.6	12.8	8.25
	2	15.2	14.3	6.25	12.5	17.0	7.22
	3	2.20	0.00	2.05	0.00	0.00	2.03
*p*-Value		0.339				0.055	
36/37	0	62.7	73.5	56.9	48.6	47.9	73.0
	1	22.4	25.0	27.8	30.0	35.7	16.8
	2	11.9	1.50	13.9	21.4	15.7	8.76
	3	3.00	0.00	1.40	0.00	0.70	1.44
*p*-Value		0.019				0.002	

F = Finely ground diet. C = Coarsely ground diet. L^+^H^−^ = Only litter. L^+^H^+^ = Litter and floor heating. L^+/−^H^−^ = Partially slatted floor. L^−^H^−^ = Fully slatted floor.

**Table 7:** Performance indices of broilers fed different feed particle sizes and reared on different floor designs (flooring × feed) from d 8 to d 36/37.

Item	Flooring								Flooring × Feed
	L^+^H^−^		L^+^H^+^		L^+/−^H^−^		L^−^H^−^		
Item	Feed								
	F	C	F	C	F	C	F	C	*p*-Value
FI (g/bird/d ± SEM)	102 ^d^ ± 0.64	111 ^ab^ ± 1.35	101 ^d^ ± 0.96	102 ^d^ ± 1.29	104 ^cd^ ± 2.53	114 ^a^ ± 2.06	108 ^bc^ ± 1.97	115 ^a^ ± 1.17	0.042
W/F ratio	2.08 ^b^ ± 0.02	2.02 ^bc^ ± 0.03	2.25 ^a^ ± 0.02	2.23 ^a^ ± 0.04	1.91 ^de^ ± 0.03	1.89 ^de^ ± 0.02	1.95 ^ce^ ± 0.01	1.97 ^cd^ ± 0.03	0.595
BW(g ± SEM) d 36/37	2328 ^bc^ ± 46.6	2439 ^ab^ ± 37.2	2243 ^d^ ± 45.8	2191 ^d^ ± 42.5	2370 ^bc^ ± 37.4	2514 ^a^ ± 43.0	2417 ^ac^ ± 51.8	2503 ^a^ ± 50.3	0.136
FCR	1.41 ^e^ ± 0.00	1.49 ^a^ ± 0.00	1.46 ^ad^ ± 0.02	1.49 ^a^ ± 0.02	1.44 ^bcde^ ± 0.00	1.48 ^ab^ ± 0.01	1.47 ^ac^ ± 0.01	1.49 ^a^ ± 0.01	0.214

^a,b,c,d,e^ Different subscripts within a row mark significant differences between the groups. F = Finely ground diet. C = Coarsely ground diet. L^+^H^−^ = Only litter. L^+^H^+^ = Litter and floor heating. L^+/−^H^−^ = Partially slatted floor. L^−^H^−^ = Fully slatted floor. FI = Feed intake. W/F = Water to feed intake ratio.

**Table 8:** Performance indices of broilers fed different feed particle sizes and reared on different floor designs (flooring, feed) from d 8 to d 36/37.

Item	Flooring					Feed		
	L^+^H^−^	L^+^H^+^	L^+/−^H^−^	L^−^H^−^	*p-*Value	F	C	*p*-Value
FI (g/bird/d ± SEM)	107 ^b^ ± 2.29	101 ^c^ ± 0.73	109 ^a^ ± 2.52	112 ^a^ ± 1.91	<0.001	104 ^b^ ± 1.11	111 ^a^ ± 1.71	<0.001
W/F ratio	2.05 ^b^ ± 0.02	2.24 ^a^ ± 0.02	1.90 ^c^ ± 0.02	1.96 ^c^ ± 0.02	<0.001	2.04 ± 0.04	2.03 ± 0.04	0.435
BW (g ± SEM) d 8	198 ± 1.78	199 ± 1.94	201 ± 2.11	202 ± 1.84	0.295	188 ^b^ ± 0.86	212 ^a^ ± 1.32	<0.001
BW (g ± SEM) d 23	1155 ± 17.8	1132 ± 17.1	1146 ± 20.2	1178 ± 18.6	0.299	1106 ^b^ ± 10.5	1200 ^a^ ± 13.6	<0.001
BW (g ± SEM) d 36/37	2383 ^a^ ± 30.5	2218 ^b^ ± 31.3	2442 ^a^ ± 29.6	2459 ^a^ ± 36.2	<0.001	2340 ^b^ ± 23.2	2413 ^a^ ± 24.1	0.023
FCR	1.45 ± 0.02	1.48 ± 0.02	1.46 ± 0.01	1.48 ± 0.01	0.155	1.447 ^b^ ± 0.01	1.490 ^a^ ± 0.01	<0.001

^a,b,c^ Different subscripts within a row mark significant differences between the groups. F = Finely ground diet. C = Coarsely ground diet. L^+^H^−^ = Only litter. L^+^H^+^ = Litter and floor heating. L^+/−^H^−^ = Partially slatted floor. L^−^H^−^ = Fully slatted floor. FI = Feed intake. W/F = Water to feed intake ratio.

**Table 9:** Dry matter content (%) in litter and pododermatitis scores of broilers fed different feed particle sizes and reared on different floor designs at d 36/37.

Item	Flooring								*p*-Value		
	L^+^H^−^		L^+^H^+^		L^+/−^H^−^		L^−^H^−^				
	Feed										
	F	C	F	C	F	C	F	C	Flooring	Feed	Flooring × Feed
DM (% ± SEM)	70.1 ^ab^ ± 1.43	67.6 ^ac^ ± 2.18	79.2 ^a^ ± 1.35	79.3 ^a^ ± 1.54	61.7 ^bcd^ ± 1.67	59.0 ^bcd^ ± 2.19	44.8 ^d^ ± 1.29	42.7 ^d^ ± 1.45	<0.001	0.461	<0.001
Pododermatitis score (Mean ± SEM, *n* = 12/box)	0.88 ^ad^ ± 0.05	0.63 ^ef^ ± 0.07	0.60 ^ef^ ± 0.07	0.74 ^cdf^ ± 0.06	0.94 ^ac^ ± 0.07	1.08 ^a^ ± 0.06	0.97 ^ab^ ± 0.05	0.82 ^bcde^ ± 0.05	<0.001	0.391	<0.001

^a,b,c,d,e,f^ Different subscripts within a row mark significant differences between the groups. F = Finely ground diet. C = Coarsely ground diet. L^+^H^−^ = Only litter. L^+^H^+^ = Litter and floor heating. L^+/−^H^−^ = Partially slatted floor. L^−^H^−^ = Fully slatted floor. DM was estimated by one pool sample (from 3 different areas in the box) for each box.
